# CAR-T cell combination therapies in hematologic malignancies

**DOI:** 10.1186/s40164-024-00536-0

**Published:** 2024-07-18

**Authors:** Delian Zhou, Xiaojian Zhu, Yi Xiao

**Affiliations:** 1Department of Hematology, Tongji Hospital, Tongji Medical College, Huazhong University of Science and Technology, Wuhan, 430030 Hubei China

**Keywords:** Combination therapy, Chimeric antigen receptor T cell therapy, Hematologic malignancies, Hematopoietic stem cell transplantation, Complete remission rate, Multiple myeloma, Malignant tumor

## Abstract

Chimeric antigen receptor-T cell therapy, a groundbreaking cancer treatment, has achieved remarkable success against hematologic malignancies. However, CAR-T monotherapy faces challenges in certain cases, including treatment tolerance and relapse rates. To overcome these challenges, researchers are investigating combining CAR-T cells with other treatments to enhance therapeutic efficacy. Therefore, this review aims to investigate the progress of research in combining CAR-T cells for hematologic malignancies. It covers the basic principles and clinical applications of CAR-T cell therapy, detailing combinations with chemotherapy, immune checkpoint inhibitors, targeted drugs, radiotherapy, hematopoietic stem cell transplantation, and other treatments. These combinations synergistically enhance the antitumor effects of CAR-T cells and comprehensively target tumors through different mechanisms, improving patient response and survival rates.

## Introduction

The principle of chimeric antigen receptor (CAR) T-cell therapy is to genetically modify T cells to recognize specific unique targets on tumor surfaces and exert cytotoxic effects [1–3]. CAR-T cell therapy has been highly successful in treating various hematologic malignancies and solid tumors [4–7]. The complete remission rate (CRR) in diffuse large B-cell lymphoma (DLBCL) is 43%, while in follicular lymphoma, it stands at an impressive 71% with sustained remissions [8]. Studies on *Idecabtagene vicleucel* for multiple myeloma (MM) involving 128 patients showed a 73% objective response rate(ORR), with 33% achieving complete remission (CR) [9]. The CRR for patients with B-cell acute lymphoblastic leukemia (B-ALL) can reach 81% [10]. Manageable adverse effects underscore the remarkable success of CAR-T products in treating hematologic malignancies, offering patients new treatment options and renewed hope. Currently, the FDA has approved six CAR-T cell products for hematologic malignancies [11–14], all with excellent treatment outcomes. CAR-T has become a reliable approach for treating various hematologic malignancies.

However, comprehensive comparative reports and studies evaluating the safety, tolerability, and efficacy of different combination therapies involving CAR-T cells for hematologic malignancies are lacking. Therefore, this review aims to investigate and analyze the progress in research concerning the combination of CAR-T cells with various treatments for hematologic malignancies. This encompasses an exploration of the fundamental principles and clinical applications of CAR-T cell therapy, along with detailed discussions on combinations involving chemotherapy, immune checkpoint inhibitors, targeted drugs, radiotherapy, hematopoietic stem cell transplantation (HSCT), and other therapeutic modalities.

## CAR-T resistance and relapse

**Fig. 1:**
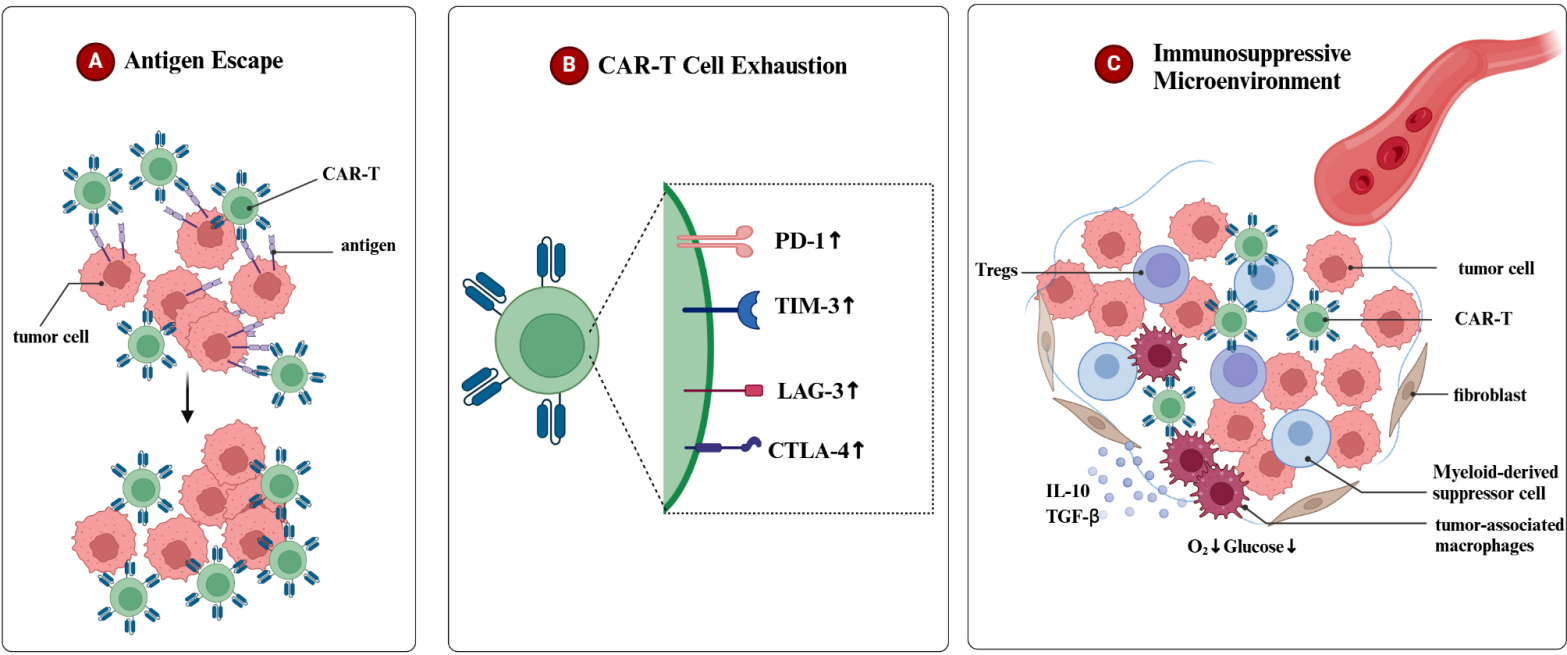
Mechanisms of CAR-T cell resistance and relapse. **(A)** Antigen Escape: Tumor cell surface antigens escape, preventing effective recognition and destruction. **(B)** CAR-T Cell Exhaustion: Increased expression of inhibitory receptors (PD-1, TIM-3, LAG-3, CTLA-4) on CAR-T cells leads to exhaustion and reduced anti-tumor function. **(C)** Immunosuppressive Microenvironment: The tumor microenvironment contains immunosuppressive cells (TAMS, Tregs, myeloid-derived suppressor cells), cytokines (TGF-β, IL-10), and the extracellular matrix. These components inhibit immune cells, weaken CAR-T cell function, and limit infiltration. Tumor cells also consume oxygen and glucose, causing nutrient deprivation and hypoxia, further reducing CAR-T cell activity

Despite CAR-T therapy demonstrating significant efficacy in some patients, it faces challenges such as tumor relapse and drug resistance in certain individuals [15–17]. Relapse rates range from 10 to 30% in B-ALL [18] and can reach 50% in DLBCL [19]. Factors contributing to this include antigen escape, CAR-T cell exhaustion, and an immune-suppressive microenvironment (Fig. 1).

### Antigen escape

During CAR-T cell therapy, tumor cells evade CAR-T cell attacks through mechanisms such as acquired mutations [20], selective splicing [21, 22], and lineage switching [23–25], resulting in mutated or reduced surface antigen targets. Common surface targets, such as CD19, CD20, CD22, and B-cell maturation antigen (BCMA), among others, are susceptible to evasion [26–28]. Clinical analysis of relapsed samples has revealed genetic mutations in CD19, found in most resistant tumor cells, potentially causing protein truncation and subsequent loss of surface antigen [29]. Several studies indicate that patients with relapsed MM treated with BCMA CAR-T therapy exhibit decreased surface expression of BCMA on tumor cells [30–32]. This antigen escape phenomenon can shorten treatment duration and patient survival while complicating therapy. Consequently, novel treatment strategies are needed to address these challenges effectively.

### CAR-T cell exhaustion

CAR-T cell exhaustion results in reduced CAR-T cell cytotoxicity, characterized by increased expression of inhibitory receptors, such as programmed cell death protein-1 (PD-1), lymphocyte activation gene-3 (LAG-3), T cell immunoglobulin and mucin domain-containing protein-3 (TIM-3), and cytotoxic T lymphocyte antigen-4 (CTLA-4), on CAR-T cell surfaces [33–38]. In treating chronic lymphocytic leukemia (CLL) with CD19 CAR-T cells, while some patients exhibit favorable responses, most do not benefit significantly from CAR-T therapy. Transcriptomic sequencing reveals that T cells in non-responsive patients upregulate programs associated with effector differentiation, glycolysis, exhaustion, and apoptosis [39]. This exhaustion significantly affects CAR-T cell functionality, suggesting that reducing exhaustion could enhance therapeutic efficacy. Research should aim to mitigate exhaustion to improve treatment outcomes.

### Immunosuppressive microenvironment

The tumor microenvironment is where CAR-T cells exert their effects. It contains numerous immunosuppressive cells (such as tumor-associated macrophages (TAMS), regulatory T cells (Tregs), and myeloid suppressor cells), suppressor cytokines (such as TGF-β and IL-10), and an extracellular matrix [40]. These elements inhibit immune-activated cells through different mechanisms, weakening CAR-T cell function and limiting their infiltration [41, 42]. Studying the influence of the immunosuppressive microenvironment on CAR-T therapy and developing strategies to counter these effects are crucial for enhancing CAR-T treatment efficacy in hematologic malignancies.

Researchers are actively exploring new approaches to address these challenges [43]. One such approach involves combining CAR-T cell therapy with other drugs, leveraging the strengths of various treatment modalities. Through this combined approach, the efficacy of CAR-T cells can be preserved and compensate for the limitations of CAR-T monotherapy, extending the survival of the patients. This comprehensive therapeutic strategy offers more holistic and effective treatment options, enhancing hope and opportunities in cancer treatment. Tables 1 and 2 present details on preclinical research and clinical studies.

**Table 1:** Preclinical research on CAR-T combination therapy

Target	Combination therapy	Type of disease	Time	Mechanism	Refs
CD19	PI3K inhibitors	CLL	2022	i. Inhibit PI3K-mediated cell apoptosis by blocking Fas signal transduction. ii. Suppress cellular exhaustion. iii .Increasing the expression of mitochondrial fusion protein MFN2.	[44]
CD19	venetoclax	R/R NHL	2022	Enhance CAR-T cell cytotoxicity.	[45]
BCMA	GSI	MM	2019	Inhibit BCMA degradation.	[46]
CS1	lenalidomide	MM	2018	i.Increase the proportion of CD8 + CAR-T cells and decrease the proportion of CD4 + CAR-T cells. ii.Promote Th1 cytokine expression in CAR-T cells and inhibit Th2 cytokines. iii.Lenalidomide improves the formation of immune synapses between CAR-T cells and tumor cells, enhancing cytotoxicity against tumor cells.	[47]
CD19	lenalidomide	DLBCL	2023	i.Polarize CD8 + CAR-T cells into CD8 + central memory cells and Th1 type. ii.Delay CAR-T exhaustion. iii.Promote CAR-T cell expansion.	[48]
BCMA	lenalidomide	MM	2019	i.Alter Th1 cell response, T cell activation, cytokine production, cell cycle control, and cytoskeletal remodeling-associated pathways. ii.In murine models, increased the number of circulating CAR-T cells in the bloodstream.	[49]

PI3K: Phosphatidylinositol 3-Kinase; CLL: chronic lymphocytic leukemia; R/R NHL: Relapsed/Refractory non-Hodgkin lymphoma; BCMA: B-cell maturation antigen; GSI: γ-secretase inhibitors; MM: multiple myeloma; DLBCL: diffuse large B-cell lymphoma

**Table 2:** Clinical research on CAR-T combination therapy

Target	Combination therapy	Disea-se	Authors	Time	Outcome	Adverse events	Clinical trials
CD19	ibrutinib	CLL	Saar Gill et al. [50]	2022	3monthsCR:44%; 48monthsOS:84%; PFS:70%	≥ 3gradeCRS:20%; ICANS:26%	NCT02640209
CD19	ibrutinib	CLL	Jordan Gauthier et al. [51]	2020	Con-ibr cohort vs. No-ibr cohort:1 year PFS :38% and 50% (*P* = 0.91)	CRS: Con-ibr cohort < No-ibr cohort	NCT01865617
CD19	lenalidomide	DLBCL	Nana Ping at al [52]	2021	C + Len cohort vs. C + cohort: 1 year OS: 100% vs. 33.3%; ORR 85.7% vs. 77.8% CR 42.9% vs. 33.3%	≥ 3gradeCRS: 43.8%;	NA
CD19	nivolumab	NHL	Yaqing Cao et al. [53]	2019	ORR:81.81%; CR:45.45%; PFS:6 months	1grade CRS:25%; 2gradeCRS:50%; ICANS:9%	NA
CD19/CD22	PD-1 inhibitor	R/R NHL	Xiangke Xin et al. [54]	2024	In CD19/CD22 CAR-Tcohort with PD-1 inhibitors vs. CAR-Talone: ORR :82.9% vs. 60% ; 2 year PFS:59.8% vs. 21.3%	≥ 3 grade CRS:13.8%, ICANS:6.2%	ChiCTR-OPN − 16,008,526 ChiCTR-OPN-16,009,847
CD19	Radiation therapy	R/R NHL	TagedPOmran Saifi et al [55]	2022	LC: bRT vs. sRT:84%vs.62%	NA	NA
CD19	Radiation therapy	DLBCL	Austin J. Sim et al. [56]	2019	ORR:81.8%; CR:45%	≥ 3 grade CRS and ICANS:27%	NA
BCMA	Radiation therapy	R/R MM	Shwetha H. Manjunath et al. [57]	2021	Group A(receive no RT < 1 year)vs. Group B(receive RT < 1 year)vs .Group C(receive bridging-RT): PR or better: 54%vs.38%vs.50%	GroupA vs. Group B vs. GroupC: G4 hematologic toxicities:61.5%vs.62.5%vs.25%; G3–4 neurotoxicity: 7.7%vs.25%vs.25%; G3-4CRS: 38.5%vs.25%vs.25%	NCT02546167
CD19/CD22	ASCT	BCL	Yang Cao et al. [58]	2021	ORR:90.5%; 2yearPFS:83.3%	≥ 3 grade CRS: 3.8%;ICANS:21%; ≥ 3 grade ICANS:5%	NA
CD19/CD22	ASCT	DHL	Jia Wei et al. [59]	2020	A cohort(CAR-T alone)ORR:75%; B cohort (CAR-T and ASCT)ORR:100%	NA	ChiCTR-OPN-16,008,526 ChiCTR-OPN- 16,009,847
CD19/CD20/CD22	ASCT	R/R CNSL	Fei Xue et al. [60]	2022	CAR-T alone ORR:44.4%;CAR-T and ASCT ORR:100%	≥ 3 grade CRS: 41%; ≥ 3 grade ICANS: 29%	NA
CD19/CD20	ASCT	R/R NHL(TP53 gene alteration)	Jia Wei et al. [61]	2022	CAR-Talone vs. CAR-T and ASCT: ORR:87.1% vs. 92.9% ; CRR: 45.2% vs. 82.1% ; 24-month OS : 56.3% vs. 89.3%	CRS:90.9%vs.94.7% ICANS:9.1%vs.19.3%	ChiCTR-OPN-16,008,526

CLL: chronic lymphocytic leukemia; CR: complete remission; OS: overall survival; PFS: progression-free survival rate; CRS: cytokine release syndrome; ICANS: immune effector cell-associated neurotoxicity syndrome; DLBCL: diffuse large B-cell lymphoma; ORR: overall response rate; NHL: non-Hodgkin lymphoma; PD-1: programmed cell death protein-1; bRT: bridging radiation therapy; sRT: salvage radiation therapy; R/R MM: Relapsed/Refractory multiple myeloma; RT: radiation therapy; ASCT: Autologous Stem Cell Transplantation; DHL: double-hit lymphoma; R/R CNSL: relapsed/refractory central nervous system lymphoma

## Combined therapeutic approaches

### Bruton tyrosine kinase inhibitors

Bruton’s tyrosine kinase (BTK) is a pivotal tyrosine kinase in the B-cell signaling pathway, regulating B-cell development, maturation, and function. BTK inhibitors are categorized as covalent [62, 63] and noncovalent. Covalent inhibitors, such as ibrutinib [64], zanubrutinib [65, 66], and acalabrutinib, [67] irreversibly inhibit the BTK enzyme. Conversely, noncovalent inhibitors, such as pirtobrutinib, reversibly inhibit the BTK enzyme [68, 69]. These inhibitors disrupt the B-cell signaling pathway, suppressing B-cell activation, proliferation, and differentiation, thereby affecting disease progression therapeutically. When combined with CAR-T therapy, BTK inhibitors enhance the efficacy of CAR-T therapy against malignant tumors by modulating T cell function and remodeling the tumor immune microenvironment.

Preclinical studies show that BTK inhibitors can enhance CAR-T cell expansion [70], reduce CD19 CAR-T cell depletion, prolong in vivo persistence, increase CD4 + and CD8 + effector memory T cells [71], promote T cell differentiation toward Th1 cells [72], decrease immunosuppressive cell populations in patients [73], and enhance cytotoxicity [74]. This effect is primarily achieved by inhibiting CD3ζ phosphorylation of CAR and downregulating genes related to the T-cell activation pathway [75], demonstrating a synergistic effect when co-administered. BTK inhibitors enhance CAR-T cell function, which is crucial for treating CLL. In in vivo animal models, combining CD19 CAR-T with ibrutinib achieves 80 ~ 100% long-term disease control. Adding ibrutinib to CAR-T cell-target cell co-cultures significantly reduces exhaustion marker expression, such as PD-1, TIM-3, LAG-3, and CTLA-4 [76], indicating CAR-T cell exhaustion reduction is key in enhancing anti-tumor efficacy in combination therapy.

Clinical studies demonstrate that administering BTK inhibitors before or concurrently with CAR-T infusion enhances clinical efficacy. Pre-treatment with BTK inhibitors reduces tumor size, decreases burden, and maintains a healthy immune microenvironment. Using CAR-T and BTK inhibitors concurrently suppresses in vivo CAR-T cell exhaustion, alleviates dysfunction, and improves expansion capacity [77]. Single-cell sequencing of 15 patients with mantle cell lymphoma (MCL) receiving combined BTK inhibitor and CAR-T therapy revealed the importance of the HSP90-MYC-CDK9 axis in this treatment strategy [78]. Further research could investigate the functional characterization of this axis and its effect on treatment responses. In a single-center study, clinical efficacy was evaluated by combining BTK inhibitors with CAR-T cells. Nineteen patients who had received ibrutinib treatment for over 6 months without achieving CR underwent CD19 CAR-T therapy. The 3-month CRR was 44%, with 15 patients showing no detectable minimal residual disease (MRD) at 6 months. The overall survival rate at 48 months reached 84%, and the progression-free survival rate (PFS) was 70%. Thirteen patients maintained CR at the follow-up endpoint. Regarding safety, grade ≥ 3 cytokine release syndrome (CRS) occurred in 20% of cases, while immune effector cell-associated neurotoxicity syndrome (ICANS) occurred in 26% [50]. These findings confirm the synergistic effect between CAR-T and BTK inhibitors, offering a novel treatment option with high and sustained remission rates. When CAR-T is administered concurrently with ibrutinib, CRS severity decreases, while ICANS occurrence remains comparable. Data indicate reduced cytokine release, including MCP-1 and IL-2a, among others, with IL-6 levels showing no significant difference from the CAR-T alone group. The 1-year PFS probabilities after CD19 CAR T-cell therapy, with or without concurrent ibrutinib, were 38% and 50%, respectively, but this difference was not statistically significant [51]. This study discovered that combining CD19 CAR T-cell therapy and ibrutinib is well-tolerated and yields lasting therapeutic effects in patients with relapsed/refractory CLL. Patients undergoing concurrent ibrutinib treatment have milder CRS severity and reduced serum cytokine concentrations during treatment. Moreover, no significant difference in PFS is observed compared to those solely on CD19 CAR T-cell therapy.

### Phosphatidylinositol 3-kinase inhibitors

The phosphatidylinositol 3-kinase (PI3K)-Akt-mTOR-c-myc signaling pathway is crucial in cancer and metabolic diseases due to its involvement in numerous protein-protein interactions and molecular crosstalk in B and T cells [79]. Studies show that adding PI3K inhibitors (PI3Ki), such as idelalisib (selective for PI3Kδ) [80] or duvelisib (a dual inhibitor of PI3Kδ and PI3Kγ), to in vitro cell experiments increases T cell numbers without hindering proliferation. Speculation suggests that PI3K inhibitors prevent PI3K-mediated cell apoptosis by blocking Fas signaling, thus inhibiting cell exhaustion [44]. During subsequent CAR-T cell manufacturing processes, incorporating duvelisib to create Duv-CART cells (CAR-T cells manufactured with duvelisib) resulted in a significant increase in CD8 + CAR-T cell number, accompanied by enhanced cytotoxicity. Bioinformatics analysis indicates that Duv-CART cells undergo epigenetic reprogramming toward stem cell-like properties. This includes upregulation of the mitochondrial fusion protein MFN2, along with increased expression of SIRT1 and TCF1/7. These changes enhance the efficacy of CAR-T cells against CLL both in vitro and in vivo [44]. Additionally, inhibiting PI3K increases stem cell central memory T cells (Tscm) and central memory T lymphocytes (Tcm) cell numbers, promoting cytotoxicity [44]. These findings underscore the crucial role of the PI3K signaling pathway in immune cell function and tumor immunity, providing valuable insights for new cancer immunotherapy strategies.

### B cell lymphoma-2 antagonists

B cell lymphoma-2 (BCL-2), a key regulator of apoptosis, belongs to the BCL-2 protein family [81], comprising two subtypes: BCL-2α and BCL-2β. The difference lies in BCL-2β lacking the transmembrane domain. This protein exerts strong antiapoptotic effects and is often overexpressed in various tumor cells, contributing significantly to cancer resistance and insensitivity [82]. Elevated BCL-2 levels promote cancer cell survival and growth, correlating with disease progression, metastasis, and adverse clinical outcomes across several malignancies [83–86], including acute myeloid leukemia (AML) and B-cell non-Hodgkin lymphoma (B-NHL). Studies indicate that BCL-2 overexpression can induce resistance to tyrosine kinase inhibitors in chronic myeloid leukemia (CML) [84]. Inhibiting BCL-2 activity or reducing BCL-2 protein levels can effectively promote apoptosis in malignant tumor cells and increase sensitivity to radiotherapy and chemotherapy [87]. Venetoclax, a BCL-2 antagonist, induces apoptosis in cancer cells overexpressing BCL-2 and is approved for patients contraindicated for frontline treatment with kinase inhibitors [88, 89]. Combining BCL-2 inhibitors with CAR-T cells is crucial for enhancing therapeutic efficacy against malignant tumors, warranting further exploration of their mechanisms of action.

Researchers investigated the effect of BCL-2 inhibitors on CAR-T cell immunotherapy in leukemia and lymphoma mouse models. They revealed, through targeted proapoptotic small molecule screening, that CAR-T cells alone achieved a killing rate of 47~63%, which increased to 75 ~ 88% when combined with BCL-2 inhibitors. This study showed that BCL-2 inhibitors enhance CAR-T cell cytotoxicity by enhancing Caspase-3/7 cleavage. However, higher doses and longer exposure times of venetoclax showed toxicity to CAR-T cells, reducing CD19 CAR-T cell numbers and inducing CAR-T cell apoptosis in the combination therapy compared to the CAR-T alone group. To address this, a venetoclax-resistant CAR-T cell was designed, targeting a specific point mutation at amino acid residue (Phe104Leu or F104L). In the MINO xenograft model, combining venetoclax with CART19-BCL-2 (F104L) achieved a 100% [45] survival rate with a cut-off of approximately 90 days. These findings underscore the potential of pairing BCL-2 inhibitors with CAR-T cell therapy and suggest strategies to mitigate venetoclax toxicity, offering valuable insights for optimizing CAR-T cell therapy further.

### γ-secretase inhibitors

The BCMA on MM cell surfaces is activated by ligands BAFF and APRIL [90–92]. BCMA expression is regulated by γ-secretase (GS), which cleaves BCMA to release soluble BCMA (sBCMA), neutralizes APRIL, and inhibits BCMA-mediated NF-κB pathway activation. This process reduces target antigen density, affecting the targeted killing ability of CAR-T cells [93, 94]. Studies using in vitro cell experiments and MM NOD/SCID mouse models showed that γ-secretase inhibitors (GSI, LY3039478) dose-dependently prevent BCMA cleavage, increasing BCMA surface expression levels on MM cells. This enhancement improves the ability of CAR-T cells to recognize tumors [46].

Based on preclinical data, a clinical study was conducted by combining BCMA CAR-T with the γ-secretase inhibitor crenigacestat (LY3039478) for treating relapsed/refractory MM. The results showed that crenigacestat increased target antigen density and was well-tolerated [95]. γ-Secretase inhibitors enhance anti-BCMA CAR-T efficacy by preventing BCMA shedding from multiple myeloma cells.

### Immunomodulatory drugs

The immunomodulator lenalidomide effectively combats hematological malignancies. Studies show that lenalidomide enhances the efficacy of CS1 CAR T cells [47] or BCMA CAR-T cells [49] in killing MM cells in vivo and in vitro when combined with CAR T cells. This effect is specifically illustrated as follows: (i) CD8 + CAR T cell subsets increased dose-dependently, while CD4 + CAR T cell subsets decreased [47]; (ii) After lenalidomide treatment on CAR T cells, autocrine cytokine IL-2 levels, which mainly determines CAR T cell efficacy and persistence, increased, and immunosuppressive Th2 cytokine levels (IL-4, IL-5, and IL-10) decreased [47]; (iii) Increasing CAR-T cell numbers [49]; (iv) Lenalidomide enhances immune synapse formation between CAR-T cells and tumors, increasing CAR-T cell lytic activity against tumors [47]. From these findings, the combination of lenalidomide and CAR-T cell therapy directly inhibits tumor-initiating cells and enhances the anti-tumor activity of CAR-T cells. Another study demonstrated that lenalidomide enhances the anti-tumor capacity of CAR-T cells by promoting CD8 + CAR-T cell differentiation into CD8 + central memory T cells and helper T cells [48], modulating the tumor microenvironment for enhanced CAR-T cell infiltration and delaying CAR-T cell depletion. Preclinical studies demonstrated that lenalidomide enhances the targeted-killing ability of CAR-T cells through various mechanisms, providing a solid foundation for further clinical trials.

A case report using piggyBac-generated CD19 CAR-T cells combined with lenalidomide to treat relapsed/refractory triple-hit DLBCL showed CR just 2 months after CAR-T cell infusion. Subsequently, they received lenalidomide maintenance therapy in the 4th month, maintaining CR for over 2 years [96]. Combining lenalidomide significantly extends the CR time. A study compared CAR-T therapy alone in nine patients with relapsed/refractory DLBCL to seven patients treated with lenalidomide maintenance therapy post-CAR-T therapy. The lenalidomide maintenance therapy group showed significantly higher survival and overall remission rates than the CAR-T therapy alone group (1-year overall survival (OS) rate 100% vs. 33.3%, ORR 85.7% vs. 77.8%, CR 42.9% vs. 33.3%). Additionally, a patient who initially responded briefly to CAR-T treatment but later relapsed achieved CR after lenalidomide therapy. PCR analysis was used to detect an increase in the CAR-T cell count [52], suggesting that lenalidomide can reverse CAR-T decline. Combining lenalidomide with CAR-T in therapy has demonstrated safe and effective clinical effects, benefiting several patients with tumors.

### Immune checkpoint inhibitors

T cells primarily harbor immune checkpoint molecules such as PD-1, CTLA-4, LAG-3, and TIM-3. These immune inhibitory molecules are crucial for preventing damage to normal tissues but can lead to T-cell dysfunction, allowing tumor cells to evade immune system attacks, a primary factor in immune tolerance [97, 98]. PD-1, expressed on various immune cells such as activated T cells, NK cells, B lymphocytes, macrophages, dendritic cells, and monocytes, is a widely studied checkpoint molecule [37]. Its ligands, PD-L1/PD-L2, are predominantly overexpressed on tumor cell surfaces. When bound, they inhibit pathways such as PI3K/protein kinase B (Akt) and RAS/MEK/ERK, block protein kinase C-theta) function and glycolysis, and impede ZAP70 phosphatidylinositol signaling [99]. This inhibition compromises T cell function, allowing tumor cells to evade T cell attacks and promoting tumor growth. Therefore, immune checkpoint inhibitors are commonly used in clinical practice to block inhibitory receptor-ligand interactions, effectively treating hematological malignancies. Combining CAR-T cell therapy with checkpoint inhibitors offers a strategy to enhance the survival rate of patients with cancer.

#### Combining CAR-T with PD-1/PD-L1 inhibitors

Cao et al. conducted a single-center study involving 11 patients with relapsed/refractory B-NHL who underwent CD19 CAR-T therapy combined with the PD-1 antibody nivolumab. The results confirmed the efficacy and safety of this combination, showing an overall remission rate of 81.81%, CRR of 45.45%, median PFS of 6 months, 75% incidence of grade 1–2 CRS, and 9% incidence of ICANS [53]. This combination demonstrates potent antitumor activity. Clinical data demonstrate that PD-1 blockade significantly enhances the anti-tumor effect. Co-expression of PD-1 and Eomes decreases on CAR-T cells, enhancing CAR-T cell expansion. This approach proves effective for patients with malignant tumors with limited CAR-T therapy response [100]. Another study retrospectively analyzed 154 patients who underwent CD19/CD22 CAR-T cell therapy with or without Autologous Stem Cell Transplantation (ASCT). It assessed the PD-1 inhibitor used for subsequent maintenance therapy. The results showed that patients receiving CD19/CD22 CAR-T therapy followed by PD-1 inhibitor maintenance had a higher ORR of 82.9% and a 2-year PFS rate of 59.8% than those not receiving PD-1 inhibitors (with an ORR of 60% and 2-year PFS rate of 21.3%). Among patients who underwent ASCT before CD19/CD22 CAR-T therapy, no significant statistical difference in ORR and 2-year PFS was observed between the two groups [54]. This suggests that PD-1 inhibitors post CD19/CD22 CAR-T therapy may benefit non-ACST patients. However, the efficacy of PD-1 inhibitors in patients with ASCT could be influenced by other factors, such as the ACST efficacy.

The timing of PD-1/PD-L1 inhibitor therapy affects CD19 CAR-T cell immunotherapy efficacy. Patients receiving durvalumab before CAR-T cell infusion show worse outcomes despite delayed and shorter CRS. Conversely, durvalumab maintenance post-CAR-T infusion enhances re-expansion, CAR-T cell numbers in the blood, and tumor cell killing [101]. Studies confirm the reliability of PD-L1 inhibitors combination therapy in terms of safety and efficacy. The combination therapy shows a 67% duration of response (DOR), exceeding the 20% DOR observed in patients receiving CAR-T therapy alone. The reduced efficacy observed with durvalumab before CAR-T therapy may be due to the early increase in soluble PD-L1 (sPD-L1) levels, which dose-dependently inhibit CAR-T cell effector function [102–104]. Clinically, careful timing of PD-1/PD-L1 inhibitor therapy is crucial for effective intratumoral lesion targeting.

#### Interfering with the PD-1 gene in CAR-T cells

CRISPR/Cas9 gene-editing technology can precisely interfere with the PD-1 gene by designing specific gRNAs to target the Cas9 protein targeted to the PD-1 gene. This induces mutations, insertions, or deletions in the genome, disrupting the function of PD-1 [105–107]. Alternative gene editing methods, such as Transcription Activator-Like Effector Nucleases and Zinc Finger Nucleases [108], can also be used to cleave and edit the PD-1 gene. RNA molecules such as small interfering RNA [109] and short hairpin RNA [110, 111] are used to inhibit gene expression by targeting PD-1 mRNA, reducing PD-1 protein levels, and swiftly inhibiting PD-1 function, although not directly editing genes.

#### Constructing CAR-T cells secreting PD-1-blocking scFv

CAR-T cells modified with PD-1-blocking scFv were used in xenograft mouse models featuring PD-L1+ expression in hematological malignancies or solid tumors [112]. This modification increases cytotoxicity, promotes cytokine secretion, and enhances in vivo antitumor effects compared to unmodified CAR-expressing [113–115]. However, a significant limitation is that this PD-1 blockade targets only CAR-T cells, leaving PD-1 on T lymphocytes in patients still immunosuppressive.

### Radiation therapy

Radiation therapy (RT) is effective for treating localized lesions in the hematologic system, including lymphoma and MM [116]. Combining CAR-T therapy with RT is a complex process but promises a treatment strategy [117, 118]. Radiotherapy induces immunostimulatory genes in lymphoma cells, enhancing signals for CAR-T cell activation and proliferation (CD70, OX40L) [119]. Gupta et al. noted that low-dose radiation (0.5–2.0 Gy) increased CD20 expression on Burkitt’s lymphoma cell lines (Raji and Daudi) with low CD20 expression [120]. This elevated CD20 expression potentially enhances CAR-T treatment efficacy by providing more targets for subsequent CAR-T cells.

Before CD19 CAR-T (Axicabtagene ciloleucel) therapy for DLBCL, using RT as a bridging treatment showed manageable toxicity. 83% of patients had reduced lymphocyte counts, leading to an ORR of 81.8%. The CRR was 45%, indicating the safety and efficacy of using RT as a bridging treatment [56]. In a retrospective analysis of 83 cases involving relapsed/refractory B-NHL patients who received RT and CAR-T therapy, researchers found that 35 patients underwent bridging RT (bRT) before CAR-T infusion, while 48 underwent salvage RT (sRT) post-CAR-T infusion. The bRT group demonstrated an 84% local control rate, significantly higher than the 62% observed in the sRT group during efficacy assessment. Additionally, the bRT group had a significantly higher remission rate than the sRT group [55]. This suggests a relationship between bRT and the local control rate in patients, while sRT offers salvage options for patients who relapse post-CAR-T infusion. DeSelm et al. documented a case involving a relapsed/refractory DLBCL patient with tumor infiltration into the skin of the right lower leg who underwent CD19 CAR-T cell therapy after local radiotherapy as scheduled. The patient had positive PET-CT results 1 month post-treatment, no toxicity at the irradiated site, and grade 1 CRS without neurotoxicity. At the one-year follow-up, the irradiated area showed no disease progression, while other areas exhibited varying degrees of recurrence or progression [121]. In a clinical trial assessing bridging radiotherapy before BCMA CAR-T therapy, radiotherapy was deemed safe and viable as a bridging treatment, demonstrating lower hematological toxicity than the no-radiation group [57]. RT can enhance CAR-T cell recognition by damaging the DNA structure of tumor cells, controlling local tumor growth, and reducing tumor burden. Furthermore, RT may reduce immune escape mechanisms in the tumor microenvironment, such as immune inhibitory factor expression, potentially inducing tumor cell apoptosis and releasing tumor-associated antigens to enhance the immune system response. This strengthens the antitumor efficacy of CAR-T cells. Therefore, selecting the appropriate timing for radiation therapy is crucial in clinical practice.

### Hematopoietic stem cell transplantation

Hematologic malignancies can achieve long-term remission or even potential cure through HSCT [122–127]. A recent study shows the efficacy of sequential CAR-T cell therapy post-HSCT. In a study of 42 patients undergoing sequential transplantation, the ORR reached 90.5%, with a 2-year PFS rate of 83.3%. Grade ≤ 3 CRS was observed in 4.8% of cases, while 21% experienced ICANS and 5% experienced severe grade 3 neurotoxicity [58]. Adverse reactions were reversible, indicating a high remission rate with a good safety profile. Additionally, researchers compared CAR-T therapy alone (Group A, eight patients) to sequential CAR-T therapy following ASCT (Group B, six patients, including two patients transitioning from Group A). The overall ORR at 3 months was 83.3%, with Group A having an ORR of 75% and Group B at 100% [59]. This study focused on relapsed/refractory double-hit lymphoma, showing that combining CAR-T with ASCT significantly increased survival, response rates, and duration of CAR-T persistence compared to CAR-T therapy alone while reducing adverse reactions and recurrence rates.

In patients with central nervous system lymphoma, combining ASCT with CAR-T cell therapy can enhance the prognosis of patients with relapsed/refractory central nervous system lymphoma. Researchers analyzed 17 such cases, with 9 receiving only CAR-T cell therapy and 8 receiving ASCT combined with CAR-T cell therapy. The efficacy assessment revealed a CRR of 100% with CAR-T cell therapy combined with ASCT and 44.4% without ASCT. Compared to standalone CAR-T cell therapy, patients undergoing combined ASCT demonstrated significantly extended PFS and OS. The incidence rates of grade ≥ 3 CRS and ICANS were 41% and 29%, respectively, with no treatment-related deaths reported [60], indicating manageable toxicity.

Patients with lymphoma that have TP53 mutations often have a worse prognosis, rendering enhancement of the efficacy in this disease subtype a key focus of clinical research. Studies show that combining CD19/22 CAR-T cell therapy with ASCT is more effective for patients with TP53 gene mutations, enhancing CRR, PFS, and OS. Clinical data from 60 patients with TP53-mutations showed that the objective response rate (ORR) was 87.1% and 92.9%, while CRR was 45.2% and 82.1% in the CAR-T alone and CAR-T combined with ASCT groups, respectively. The 24-month OS rates were 56.3% vs. 89.3% [61], indicating a significant improvement in patient outcomes with this combination therapy.

Combining CAR-T cell therapy with HSCT can be an effective and safe strategy for treating certain hematologic malignancies, leading to enhanced treatment outcomes and prognosis for specific patient subgroups. While some patients may experience adverse reactions, they are typically manageable, indicating the good safety profile of the combination therapy. However, further clinical research is necessary to establish the optimal treatment approach for different types of tumors and individual patients.

**Fig. 2:**
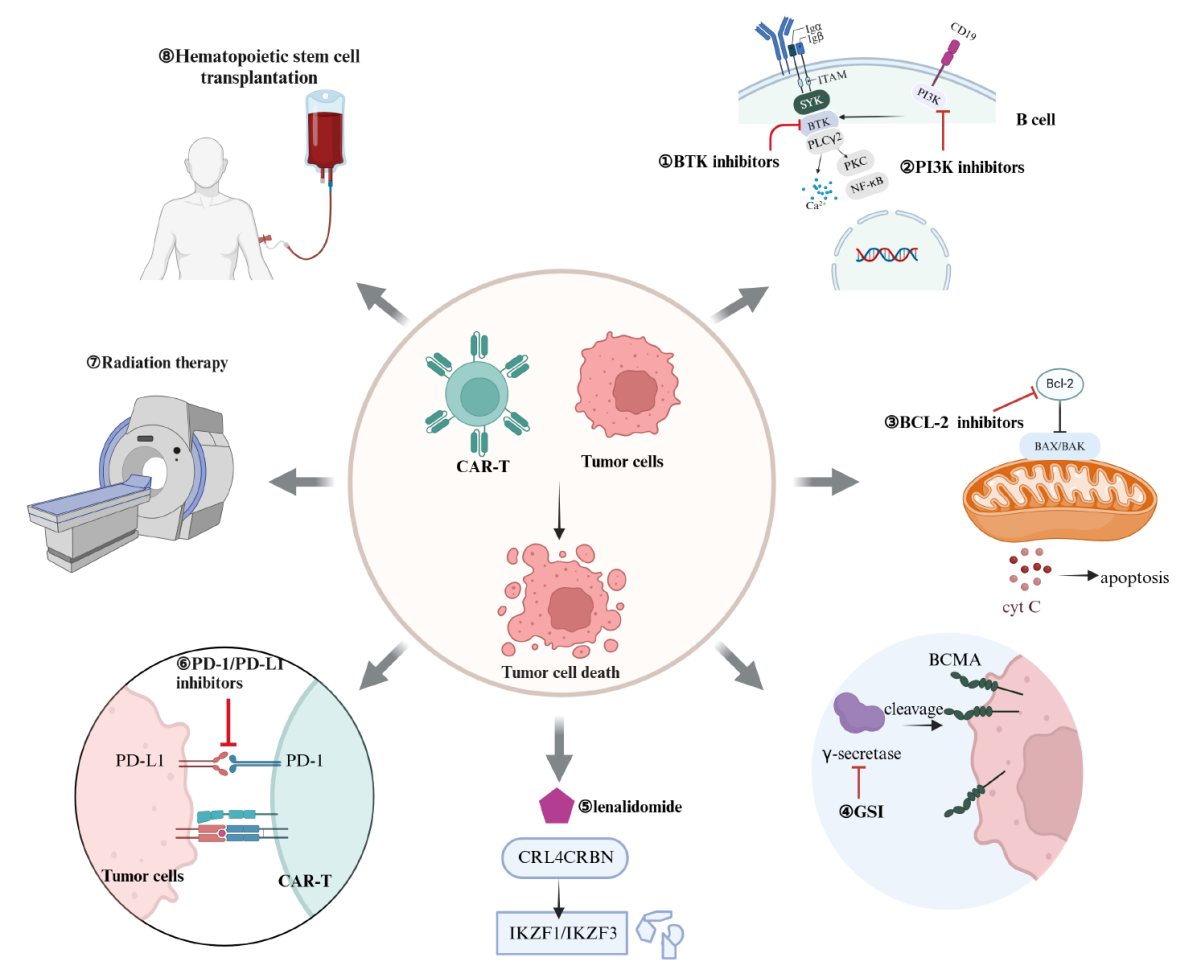
Combination Therapies with CAR-T Cells. ①Combining CAR-T cells with BTK inhibitors inhibits BTK in the B-cell signaling pathway, reducing malignant B-cell proliferation. ②Combining CAR-T cells with PI3K inhibitors interferes with tumor cell growth and survival by inhibiting the PI3K signaling pathway. ③Combining CAR-T cells with BCL-2 inhibitors promotes tumor cell apoptosis by inhibiting the BCL-2 protein. ④Combining CAR-T cells with GSI prevents the shedding of BCMA from the surface of tumor cells by inhibiting γ-secretase. ⑤Combining CAR-T cells with Lenalidomide recruits the E3 ubiquitin ligase CRL4CRBN, inducing the degradation of IKZF1 and IKZF3. ⑥Combining CAR-T cells with PD-1/PD-L1 inhibitors blocks the PD-1/PD-L1 signaling pathway to counteract the tumor cells’ immune evasion mechanism. ⑦Combining CAR-T cells with Radiotherapy directly kills tumor cells, enhancing the effectiveness of CAR-T therapy. ⑧Combining CAR-T cells with Hematopoietic Stem Cell Transplantation rebuilds the patient’s immune system to support the sustained anti-tumor activity of CAR-T cells.

With thorough research and continuous advancements in CAR-T technology, combining CAR-T with other treatments has expanded its applicability and increased treatment options for patients with cancer. Research on various combination therapy strategies in the evolving treatment landscape is bringing new hope for clinical practice and patient well-being (Fig. 2).

## Conclusion

CAR-T cell therapy, an emerging cancer treatment modality, shows great promise in treating hematologic malignancies. However, some limitations, such as patient tolerance and post-treatment relapse, are associated with this strategy. To address these limitations, researchers are investigating combining CAR-T cell therapy with other treatment modalities to enhance therapeutic efficacy and reduce side effects. Several studies on combination therapies have significantly advanced clinical effectiveness and reduced relapse rates in patients with relapsed/refractory. Detailed insights into combining CAR-T cells with chemotherapy, immune checkpoint inhibitors, targeted drugs, radiotherapy, HSCT, and other treatments are provided in this study. These combination therapies enhance the anti-tumor effects of CAR-T cells and comprehensively target tumors through diverse mechanisms, improving patient response and survival rates. This offers novel insights and strategies for enhancing the clinical application of CAR-T cell therapy. Comparative reports on the safety and tolerability of different combination therapies are lacking, which is a potential starting point for future research to identify the most suitable combination therapy. Furthermore, optimizing protocols for CAR-T combination therapy is necessary to accommodate the diverse needs of patients, including timing, dosages, and post-treatment care. Further clinical studies are needed to validate the safety and efficacy of these combination treatment strategies. With continuous advancements in science, technology, and clinical experience, CAR-T cell combination therapy is expected to play an increasingly vital role in the future, improving treatment outcomes and survival prospects for patients with hematologic malignancies.

## Data Availability

No datasets were generated or analysed during the current study.

## Abbreviations

CAR: Chimeric antigen receptor
CRR: Complete remission rate
DLBCL: Diffuse large B-cell lymphoma
MM: Multiple myeloma
ORR: Objective response rate
CR: Complete remission
B-ALL: B-cell acute lymphoblastic leukemia
HSCT: Hematopoietic stem cell transplantation
BCMA: B-cell maturation antigen
PD-1: Programmed cell death protein-1
LAG-3: Lymphocyte activation gene-3
TIM-3: T cell immunoglobulin and mucin domain-containing protein-3
CTLA-4: Cytotoxic T lymphocyte antigen-4
CLL: Chronic lymphocytic leukemia
TAMS: Tumor-associated macrophages
Tregs: Regulatory T cells
BTK: Bruton tyrosine kinase
MCL: Mantle cell lymphoma
MRD: Minimal residual disease
PFS: Progression-free survival rate
CRS: Cytokine release syndrome
ICANS: Immune effector cell-associated neurotoxicity syndrome
PI3K: Phosphatidylinositol 3-kinase
PI3Ki: PI3K inhibitors
Tscm: Stem cell central memory T cells
Tcm: Central memory T lymphocytes
BCL-2: B cell lymphoma-2
AML: Acute myeloid leukemia
B-NHL: B-cell non-Hodgkin lymphoma
CML: Chronic myeloid leukemia
GS: γ-secretase
GSI: γ-secretase inhibitors
sBCMA: Soluble BCMA
OS: Overall survival
ORR: Overall response rate
ASCT: Autologous Stem Cell Transplantation
DOR: Duration of response
sPD-L1: Soluble PD-L1
scFv: Single-chain variable fragments
RT: Radiation therapy
bRT: Bridging radiation therapy
sRT: Salvage radiation therapy
